# 

**Published:** 1819-01

**Authors:** 


### THE LONDON MEDICAL AND PHYSICAL JOURNAL;

CONTAINING

ORIGINAL CORRESPONDENCE of EMINENT PRACTITIONERS,

AND THE

EARLIEST INFORMATION ON SUBJECTS CONNECTED WITH

*MEDICINE, SURGERY, CHEMISTRY, PHARMACY, BOTANY, AND NATURAL HISTORY.*

---

*Successively conducted by*

T. BRADLEY, M.D.

A. F. M. WILLICH, M.D.
R. BATTY, M.D.
A. A. NOEHDEN, M.D.
J. ARNEMAN, M.D.
J. P. PFAFF, M.D.
J. ADAMS, M.D.
W. SHEARMAN, M.D.
W. ROYSTON, Esq.

SAMUEL FOTHERGILL, M.D.

AND

WILLIAM HUTCHINSON, Esq.
SURGEON;

ASSISTED BY EMINENT GENTLEMEN, IN THE VARIOUS BRANCHES OF THE PROFESSION, AS WELL IN ENGLAND AS IN FRANCE, GERMANY, ITALY, AND THE UNITED STATES.

---

VOL. XLI.
FROM JANUARY TO JUNE, 1819.

5147

Et quoniam variant morbi, variabimus artes;

Mille mali species, mille salutis erunt.

---

*LONDON:*

PRINTED FOR THE PROPRIETORS,
BY J. AND C. ADLARD, 23, BARTHOLOMEW-CLOSE;
PUBLISHED BY J. SOUTER, 73, ST. PAUL'S CHURCH-YARD; AND
MAY BE HAD OF ALL BOOKSELLERS.

W1
LO 31

### ANALYTICAL TABLE OF THE CONTENTS

OF

THE FORTY-FIRST VOLUME

OF THE

LONDON MEDICAL AND PHYSICAL JOURNAL.

---

### DESCRIPTIVE ANATOMY.

Page.

TREATISE on . . . . . . . . . . 228

MUSCULAR SYSTEM.—Description of the origin and development of the cremaster muscle . . . . . . . . 7

NERVOUS SYSTEM.—Determination of the existence of the spheno-palatine ganglion in man, its description, and connections . 6

OSSEOUS SYSTEM.—Precise determination respecting the formation of the sphenoidal sinuses . . . . . . . 8

### PHYSIOLOGY.

VITAL PHENOMENA, *in general*.—Remarks on . . . 20, 75

On the mode in which they are influenced by external agents 142, 173, 204, 243, 254, 516

NERVOUS AND SENSITIVE SYSTEMS.—Opinions respecting the general and local development of nervous energy . . 20, 24, 26

The brain and spinal marrow apparently that of sensitive and animal life . . . . . . 21, 23, 24, 25, 26, 74

The nervous ganglions apparently that of nutritive or organic life . . . . . . 20, 24, 26, 28, 74

Influence of the different nerves, and their centres, on each other 21, 22, 23, 24, 25, 26, 27, 29, 74, 130, 172, 173, 175, 243, 244, 246, 247, 338, 436

Consequences of the duplication of the nervous system of animal life . . . . . . . . . . 37

NUTRITIVE FUNCTIONS (*organic*, of BICHAT).

*General observations respecting them*.—Existence of them in a fœtus without either brain or spinal marrow . . . . 20

Cause of cessation of, on destruction of the brain in extra-uterine life . . . . . . . . . 21

*Appetite for, and mode of obtaining, the food*. Observations on Anthropophagism . . . . . . . 215

*Mastication* . . . . . . . . .

*Insalivation* . . . . . . . . .

*Deglutition* . . . . . . . . .

*Digestion*. General view of the process of . . . 105, 107

*Absorption* . . . . . . . . .

*Circulation*. Its continuance in a fœtus without either brain or spinal marrow . . . . . . . . . 20

Influence of distinct portions of the nervous system on it 21, 130

Various characters of arterial pulsation . . . 464

The agency of the capillary arteries . . . 418, 507

*Respiration*. Its existence essential, in extra-uterine life, for the development of nervous influence . . . . 22

Causes of, and of its cessation on destruction of the eighth pair of nerves . . . . . . . . . *ib.*

Page.

*Assimilation.* Not essentially dependent on nervous influence, since it precedes the existence of nerves . . . 28
Observations respecting the mode of reparation of parts . 92
*Secretion.* Change of the natural colour of that of the dermoid organ . . . . . . . 30, 141, 215
Influence of different species of food on the composition of the urine . . . . . . . . . 110
Phosphorescent, instances of . . . . . 452, 540
*Excretion.* Cerebral influence necessary to effect it in some instances 26
ANIMAL FUNCTIONS.—*General observations on* . . . 22
*Sight* . . . . . . . . .
*Hearing.* General observations on . . . . 281
*Smell* . . . . . . . .
*Taste* . . . . . . . .
*Touch.* Remarkable delicacy of, in a blind young female . 126
*Sensations* or *Perceptions, in general, and their results.* May exist and induce movements without consciousness . . 24
Consequences of those derived from the connexion of the ganglionic system of nerves with the brain . . . 26, 175, 215
General view of the causes of . . . . . 26
*Movements.* May be produced by the influence of the ganglionic system of nerves on the extremities of those of the brain and spinal marrow, without the intervention of the cerebral centre, in the fœtus, but not after birth . . . 33, 27
May be produced by the influence of the ganglionic system of nerves on the brain without volition . . . . . 23
Curious instance of abolition of the power of producing 31, 88
*Sleep.* Reference to some interesting observations and reflections on its cause . . . . . . . . . 322
*Voice* . . . . . . . .
GENERATIVE FUNCTIONS.
*Preparatory to the act of generation.* Results of the sympathy existing between the different organs subservient to them . 29
*Generative act*, when prolific, causes a rupture of the corpora lutea and escape of ova . . . . . . . 357
*Ordinary process of generation, and accidental deviations from it.*
Communication of vessels in the placentas of twin-fœtuses . 31
Instance of expulsion of one fœtus, and retention of another, of twins . . . . . . . . . . 222
Existence of a fœtus within the cavity of the peritoneum in a boy 311
Functions of the uterus during pregnancy . . . 352
Superfœtation, cases of . . . . . . 404, 512
Observations relative to the duration of the periods of, in various animals . . . . . . . . 537
*Sexual characteristics* . . . . . .
*Lactative function* . . . . . . .
PECULIARITIES IN THE FUNCTIONS AT THE DIFFERENT PERIODS OF LIFE.
*Fœtal life.* May exist without brain or spinal marrow . . 20
*Infancy.* Not so intimate a sympathy between the different nerves and their centres during this epoch as in the further development of the organization . . . . . . . 21
*Puberty* . . . . . . . .
*Manhood* . . . . . . . .
*Old age* . . . . . . . .

### COMPARATIVE ZOOLOGY.

Anatomical and physiological account of the Medicinal Leech . 312

### PATHOLOGY.

Page.
GENERAL OBSERVATIONS . . . 170, 174, 243, 244, 246
ABSCESS, *Psoas*, remarkable case of . . . . . 320
AMAUROSIS, case of, induced by terror . . . . 466
ANEURISM, case of, by anastomosis . . . . . 224
ANUS, observations on spasmodic constriction of . . 69, 459
APOPLEXY, arises from obstruction of the circulation in the brain . 32
Its connexion with diseases of the lungs and heart . . 34
Similar to syncope . . . . . . . *ib.*
Dependent in some cases on inflammation of the brain . 321
Information respecting it furnished by anatomy . . 10
CANCER. Observations on its nature and origin . . . 467
CHICKEN-POX. Severe cases of disease resembling it . . 39
COMA. That of dormant hybernating animals is accompanied with very slow respiration . . . . . 31
Not always accompanied with change of structure of the brain 32
Induced by interruption of the circulation in the brain . 35
COW-POX. Observations relative to its prophylactic powers against small-pox . . . . . . 39, 451
Prophylactic against the plague . . . . 189
Observations and remarks adverse to its use . . . 271
CRANIUM, singular disease of . . . . . 303
DROPSY, cases of, with remarks . . . . . 410
DYSENTERY. Observations on its inflammatory nature . . 509
DYSPEPSIA, frequently dependent on chronic inflammation of the stomach . . . . . . . . 509
ERETHISM, *mercurial*, cases of . . . . . 92, 224
FEVER. The identity of character of all its forms . . 47, 49
Its connexion with inflammation of some organ . 48, 49, 173, 247, 337, 435, 505
Opinions in favour of the existence of idiopathic fever 503, 509
Its origin from deficient and vitiated aliment . . 49, 50
Its origin from atmospheric influence and from contagion 50, 51, 52, 485, 510
The nature and origin of *yellow fever* (from marsh miasmata) 53, 151
*Spotted*, of North America, history of . . . . 323
*Intermittent*, nature of . . . . . . . 433
*Puerperal*, its nature and origin . . . . 438
Information respecting it furnished by anatomy 17, 251, 506, 507
FISTULÆ, observations on . . . . . . 251
FUNGUS HÆMATODES, case of . . . . . 388
HEART, *rupture of* . . . . . . . 446
HYDROCELE. Circumstances attendant on its cure, and some hints respecting its cause . . . . . . . 67
HYDROPHOBIA, cases of, terminating fatally . . 55, 450
Observations respecting its nature and origin . . 81
Information respecting it furnished by anatomy . . 14
HYDROTHORAX, case of . . . . . . 215
ICTHYOSIS, case of . . . . . . . 149
ISCHURIA, case of . . . . . . . 206
INFLAMMATION, observations on, general and particular . 36, 173, 244, 247, 393, 416
INTESTINES, case of strangulation of, from rupture of the mysentery 132
Catarrhal inflammation of . . . . . 331
JOINTS, Mr. Brodie's classification, and history, of the diseases of . 156
Professor Rust's observations on the most frequent diseases of, 183, 341
LEPROSY, *tubercular*, case of . . . . . . 267
LUXATIONS. Case of complete luxation of the tibia from the femur anteriorly . . . . . . . . 64
Of the epiphyses of the bones . . . . . 66

| | Page. |
|---|---|
| Mania, observations on, and its origin from disease of the gastric system | 174 |
| Marasmus, observations on . . . . . . | 236 |
| Nævi Materni, observations on a peculiar species of . . | 222 |
| Ophthalmia, observations on . . . . . | 511 |
| Paralysis, remarkable case of . . . . . | 88 |
| Pancreas, enlargement of, with cases . . . . | 408 |
| Phlegmasia Dolens, case of, terminating favourably . . | 200 |
| Phthisis. Enquiry respecting the influence of soil and climate in the production of . . . . . . . . | 229 |
| Pleurisy, case of . . . . . . . | 134 |
| Scurvy. Observations respecting its nature and origin . . | 515 |
| Small-pox. Account of, and reflections respecting, an epidemic disease resembling it . . . | 38, 94, 288, 457 |
| Swine-pox. Its characteristic . . . . . | 41 |
| Tetanus. Its ordinary character, and origin . . | 77, 78, 79 |
| Case of, which terminated favourably . . . . | 449 |
| Information respecting it furnished by anatomy . | 11, 78 |
| Veins, *varicose*, observations on . . . . . | 67 |
| Inflammation of . . . . . . . | 492 |
| Venereal Diseases, various, resembling syphilis . | 59, 62, 63, 64 |
| Syphilis now of but rare occurrence . . . | 59, 60 |
| Worms, *intestinal*. Cases in which they perforated the stomach and intestines . . . . . . . | 16 |
| Observations relative to their generation . . . | 497 |

### PATHOLOGICAL ANATOMY.

| | |
|---|---|
| General observations respecting its utility and application in medical science . . . . . . . . | 9 |
| Brain. Formation of a cyst in it, around coagula of extravasated blood . . . . . . . . . | 11 |
| Peculiar disorganization of it, in which portions of it were changed into a pultaceous mass . . . . . | 12 |
| Heart. Case of lesion of, with a pervious state of the foramen ovale | 364 |
| Rupture of . . . . . . . . . | 446 |
| Hydatids, *of the Uterus*. Real nature and description of . . | 15 |
| Intestinal Canal. Perforation of it by worms . . . | 16 |
| Nerves. Enlargement of the sympathetic nerve in diabetes . | 14 |
| Polypus *in the Colon*, description of . . . . | 407 |
| Spinal Marrow. | |
| Disease of, a frequent, if not general, cause of tetanus . | 13 |
| suspected as the cause of hydrophobia . . | *ib.* |
| present in typhous fever . . . . | 14 |
| trismus nascentium . . . | *ib.* |
| in tetanus . . . . | 78 |

### NOSOLOGY.

| | |
|---|---|
| Proposal for the arrangement of diseases in classes, according to the portion of the nervous system principally affected . . | 74 |
| Miscellaneous reflections . . . . . . | 193, 201 |

### SEMIOLOGY.

| | |
|---|---|
| Relative to Groups of Symptoms, or Diseases. | |
| Of the gastric system in general . . . . | 57 |
| Hydrophobia . . . . . . . . | 81 |
| Joints, various diseases of . . . . | 157, 343 |
| Luxation of the knee-joint . . . . . | 66 |
| Pancreas, chronic enlargement of . . . . | 408 |

Page.

Of pocks, *chicken* . . . . . . 39, 40, 41

*cow* . . . . . . 39 *et seq.*

. . *small* . . . . . . *ib. et seq.*

. . *swine* . . . . . . *ib. et seq.*

Tetanus . . . . . . . . 79

Venereal diseases . . . . . . 61 *et seq.*

Yellow-fever . . . . . . . 154

RELATIVE TO PARTICULAR FUNCTIONS.

Various critical evacuations . . . . . . 244

### HYGIENE.

Relative to contagious fever . . . . . . . . 269

### THERAPEUTICS.

*Relative to* Amaurosis . . . . . . . 466

Aneurism . . . . . . 123, 148, 189

Asthma . . . . . . . . 116

Cancer . . . . . . . 84, 467

Dropsy . . . . . . . 412, 536

Dysentery . . . . . . . 509

Erethismus, *mercurial* . . . . . 93, 224

Fever . . . . 155, 339, 421, 432, 434

Gonorrhœa . . . . . . . 537

Hydrocele . . . . . . . 67, 148

Hydrophobia . . . . . . 55, 82

Hydrothorax . . . . . . . 315

Joints, the diseases of . . . . . 159, 318

Ichuria . . . . . . . . 206

Mania . . . . . . . 128, 175

Ophthalmia . . . . . . . 511

Scurvy . . . . . . . . 524

Stricture, of the anus . . . . . 69, 459

Tetanus . . . . . . 80, 212, 449

Veins, varicose . . . . . . 67, 492

Venereal diseases . . . . . . 60 *et seq.*

### CHIRURGERY.

*On the Operations relative to* Abscesses . . . . . 349

Amputation . . . . . . . 306

Aneurism . . . . . . 208, 377, 495

Castration . . . . . . . 212

Circumcision . . . . . . . 307

Fistula lachrymalis . . . . . . 267

Hydrocele . . . . . . . . 67

Lithotomy . . . . . . . . 358

Membrana tympani, puncture of . . . . 281

Paracentis, of the thorax . . . . . 138, 315

Parturition . . . . . . . 146, 512

Rhinoplastick art . . . . . . . 71

Stricture, of the anus . . . . . . 69, 459

Tumors, in the neck . . . . . . . 406

Varicose veins . . . . . . 67, 492

### OBSTETRICISM.

Reflections on some means used to accelerate parturition . . 301
Observations on the use of abdominal pressure in parturition . 121, 300
Turning of the fœtus effected in a case of rupture of the uterus, with a favourable termination . . . . . 319

Page.
Physiological view of the function of parturition . . . 332
Observations on the consequences of the more severe states of puerperality . . . . . . . . 350
Management of cases of extra-uterine fœtation . . . 512

### CHEMISTRY.

Historical Sketch of, as applied to Medicine . . . . 97

Its application to Physiology and Pathology . . . . 103

Composition and formation of the chyme, chyle, and blood . . 104

Composition and formation of urinary calculi . . . 106, 109

On the tests for arsenic . . . . . . . 136

### MATERIA MEDICA, PHARMACY, AND MEDICAL BOTANY.

TREATISES ON MATERIA MEDICA, &c.
Codex Medicamentarius of Paris . . . . . 112
Tercer año Medico-Clinico de la real Escuala de Med. Prat. de Barcelona, by Dr. SALVA . . . . . . *ib.*
Vegetable Materia Medica of the United States of North America, by Dr. BARTON . . . . . . 113
American Medical Botany, by Dr. BIGELOW . . . . *ib.*
Traité de Matiere Medicale, &c. par M. SCHWILGUE . *ib.*
Formulaire Magistrale, &c. par Dr. Cadet de GASSICOURT . *ib.*
London Dispensatory, by Mr. TODD THOMSON . . . 114
Supplement to the Pharmacopœias, by Mr. GRAY . . *ib.*
INDIVIDUAL OBSERVATIONS ON MATERIA MEDICA, &c.
Antimony, tartarised, its febrifuge properties . . . 114
Arsenic, its utility in intermittent fever . . . 115
Cassia Marilandica, botanical history of . . . 213
Catheter, new form of . . . . . . 358
Colchicum, its diuretic properties . . . . 115
Digitalis, its mode of action . . . . . 421
Galvanism, its application as a therapeutical measure . . 116
Moxa, its medical utility . . . . . 119
Nux vomica, its utility in paralysis . . . . *ib.*
Silver, nitrate of, its good effects in various diseases . . *ib.*
Toddalia, a new febrifuge bark . . . . . *ib.*
Transfusion of blood, recommended as a medical measure . 150
Turpentine, oil of, its use in some diseases of mucous membranes 406

### MISCELLANEA.

Lectures, CROONIAN, for the year 1818 . . . . 92
Observations on Phosphorescent Animals . . . 452, 540
Medical Statistics . . . . . . 277, 538
Observations on Surgical Education . . . . . 369
Reports of Diseases . . . 95, 190, 279, 366, 455, 543
Prize Questions . . . . . . 190, 361
Meteorological Tables . . . 96, 191, 280, 367, 456, 544
Monthly Catalogues of Books . . . . 278, 366, 454, 542
Essay on the Proximate Causes of the Mechanical Phenomena of the Universe . . . . . . . . 185

### NAMES OF AUTHORS,

*Of whose Works, Observations, &c., a detailed Account, or general View, is given in the*

FORTY-FIRST VOLUME OF THE

### LONDON MEDICAL AND PHYSICAL JOURNAL.

---

Page.

ABERCROMBIE, Dr. J. of *Edinburgh*, observations on Apoplexy, Paralysis, and Coma . . . . . . . . 31
ALBERS, Dr. of *Bremen*, case of Popliteal Aneurism cured by Compression . . . . . . . . 149
ALIBERT, Dr. and Professor, of *Paris*, case of tubercular Leprosy . 267
ARCHER, Dr. of *Dublin*, case of Paracentesis of the Thorax . . 315
ASBURY, Mr. of *Enfield*, observations on the Puncture of the Membrane of the Tympanum . . . . . . 281
ASTBURY, Dr. of *Barlaston*, cases of Mercurial Erethism . . 92
AYRE, Dr. of *Hull*, Treatise on Marasmus . . . . 236
BACHE, Dr. of *Philadelphia*, on the Effects of varied Temperature on the Animal Economy . . . . . 259
BALDY, Mr. of *Plymouth*, case of Fungus Hæmatodes . . 388
BALFOUR, Dr. of *Edinburgh*, on the Medicinal Properties of Nitrate of Silver . . . . . . . . 194
BALME, Dr. of *Lyons*, Treatise on Scurvy . . . . . 515
BARRY, Dr. MILNER, of *Cork*, on the Origin of Intestinal Worms . 497
BATEMAN, Dr. of *London*, observations on Fever . . . 47
Case of Mercurial Erethism . . . . . . 224
BENT, Dr. of *Derby*, observations on the Epidemic Varioloid Disease of Derby . . . . . . . . 38, 477
BERARD, Dr. of *Montpellier*, Treatise on Varioloid Diseases . . 45
BLUNDELL, Dr. of *London*, on the Transfusion of Blood . . 150
BOND, Mr. of *Norwich*, case of Ligature of the external Iliac Artery 123
BOSTOCK, Dr. of *London*, remarkable case of Paralysis . . 88
BOYER, M. LE BARON, of *Paris*, on Spasmodic Constriction of the Anus . . . . . . . . . . 69
BRESCHET, Dr. of *Paris*, observations on Fistulæ . . . 257
BRICHETEAU, Dr. of *Paris*, observations on Apoplexy . . 11
BRODIE, Mr. of *London*, Treatise on Diseases of the Joints . . 155
BROUSSAIS, Dr. of *Paris*, observations on the Functions of the Nervous System, &c. . . . . . . . . . 22
His general Pathological Doctrine . . 167, 243, 337, 432
BROWN, Mr. of *Musselburgh*, on Vaccination . . . . 272
CARMICHAEL, Mr. of *Dublin*, on Venereal Diseases . . . 60
On the Treatment of Varicose Veins . . . . . 67
Extirpation of a Tumor in the Neck . . . . . 406
On Inflammation of Veins, and on the application of a Ligature to the Femoral Artery . . . . . . 492
CHAMPION, Dr. of *Bar-le-Duc*, cases of Separation of the Epiphysis of the Long Bones . . . . . . . 66
CHAPMAN, Mr. of *Windsor*, case of Expulsion of a Fœtus, whilst another was retained in the Uterus . . . . . 222
CLARK, Dr. of *Philadelphia*, on the use of Colchicum in Dropsy . 115
CLAUBRY, Dr. DE, of *Paris*, on some unusual Effects of Worms . 16
CLOQUET, Dr. HIPPOLYTUS, of *Paris*, on the Spheno-Palatine Nervous Ganglions . . . . . . . . . 5
CLOQUET, Dr. JULIUS, of *Paris*, on the Cremaster Muscle . . 6

Page.

Collier, Mr. of *London*, on the Opinions of the Ancients respecting the Functions of the Arteries . . . . . 197
Crampton, Dr. John, of *Dublin*, Clinical Report on Dropsies . 410
Curtis, Mr. of *London*, on the Treatment of Puriform Discharge from the Ear . . . . . . . . 397
Duncan, Dr. jun. of *Edinburgh*, observations on Fever . . 49
Finot, Dr. of *Luxeuil*, cases of Paralysis cured by Nux Vomica . 182
Fischer, Dr. of *Hildburghausen*, case of Rupture of the Heart . 446
Gaitskell, Dr. of *Bath*, Treatise on Catarrhal Inflammation of the Intestines . . . . . . . . . . 331
Gasc, Dr. of *Paris*, on the use of Arsenic in Intermittent Fever . 115
Graefe, Prof. of *Berlin*, on the Rhinoplastick Operation . . 71
Grattan, Dr. of *Dublin*, on Inflammation . . . . . 36
On the Treatment of Fever . . . . . . 416
Hale, Dr. of *Maine in the United States*, Treatise on Spotted Fever . 323
Hall, Dr. Marshall, of *Nottingham*, Treatise on the Mimoses . 57
Hall, Dr. of *Berwick*, case of Tetanus . . . . 449
Hamilton, Mr. Wm. of *Ipswich*, Obstetrical Observations . . 121
Hennen, Mr. of *Edinburgh*, on Varioloid Diseases . . . 38
On Venereal Diseases . . . . . . . . . 60
Hill, Mr. Nesse, of *Chester*, on the Treatment of Mania . . 128
Home, Sir Everard, of *London*, Croonian Lectures . . . 92
On the Corpora Lutea . . . . . . . 357
Hume, Mr. of *London*, on the Tests for Arsenic . . . 131
James, Mr. of *Hereford*, case of Fever . . . . . 130
Johnson, Dr. of *Dundee*, case of Hydrophobia . . . 450
Jones, Mr. of *London*, case of Strangulated Intestine . . 132
Amaurosis . . . . . . . . . 466
King, Mr. of *South Carolina*, Treatise on Extra-uterine Fœtation, &c. 512
Kinglake, Dr. of *Taunton*, Nosological Strictures . . . 300
Klapp, Dr. of *Philadelphia*, Essay on Temulent Diseases . . 174
Kurzmann, Dr. of *Berlin*, Treatise on the Leech . . . 312
Lallemand, Dr. of *Paris*, Physiological Observations . . 20
Lanyon, Mr. of *Lostwithiel*, case of Pleurisy . . . 450
Lavalette, Dr. of *Paris*, observations on Luxation of the Knee-joint 65
Lavit, Dr. of *Montpellier*, Treatise on Varioloid Diseases . . 45
Lescure, Dr. of *Paris*, observations on the Utility of Nux Vomica in Paralysis . . . . . . . . . . 181
Locher, Dr. of *Zurich*, case of Hysterotomy . . . . 146
Macartney, Dr. and Prof. of *Dublin*, Pathological Observations on the Bodies of the Patients of Fever . . . . . 17
Maclean, Dr. of *Edinburgh*, case of Ichuria . . . . 206
Magendie, Dr. and Prof. of *Paris*, Treatise on Urinary Calculi . 109
Mangili, Dr. and Prof. of *Padua*, Lecture on the Formation of Uterine Hydatids . . . . . . . . . . 15
Memoir on the Hybernation of Animals . . . . 31
Mansford, Mr. of *Frome*, Treatise on the Influence of Climate in the Production of Phthisis . . . . . . 229
Marson, Mr. of *Worksop*, Obstetrical Observations . . 300
Martin, Mr. of *Pulborough*, case of Icthyosis . . . . 149
Meckel, Dr. and Prof. of *Halle*, Treatise on Pathological Anatomy 364
Mehlis, Dr. of *Gottingen*, Treatise on the Physiological and Pathological Consequences of the Duplication of the Nerves of Animal Life . . . . . . . . . . 37
Monro, Dr. and Prof. of *Edinburgh*, Treatise on Small-pox . . 46
Musgrave, Dr. of *Antigua*, observations on Yellow Fever . . 53
Nichol, Dr. of *Ludlow*, case of Angina Pectoris . . . 507
O'Brien, Dr. of *Dublin*, observations on Fever . . 50, 508
Oven, Van, Mr. of *London*, Reflections on Surgical Education . 369
Parkinson, Dr. of *London*, Nosological Strictures . . . 208
Observations on the Nature of Cancer . . . 407
Parkinson, Mr. James, of *London*, observations relative to the Prevention of Fever . . . . . . . 269

Page.
**Percival**, Dr. of *Dublin*, observations on Enlargement of the Pancreas 408
**Pew**, Dr. of *Sherborne*, observations on Varioloid Diseases . . 155
**Philip**, Dr. **Wilson**, of *Worcester*, various Physiological Reflections, 20, 107
On the Medical Use and Application of Galvanism . . 116
**Phillips**, Sir **Richard**, of *Holloway*, Essays on the proximate Mechanical Causes of the Phenomena of the Universe . . 185
**Power**, Mr. of *Market Bosworth*, Treatise on Midwifery . . 322
**Prout**, Dr. of *London*, observations on Sanguification . . 105
**Rayner**, Mr. of *Warwick*, case of Phlegmasia Dolens . . 200
**Reid**, Dr. of *Dublin*, Treatise on Tetanus and Hydrophobia . 73
**Renwick**, Dr. of *Liverpool*, observations relative to the Sense of Touch, &c. . . . . . . . . . 125
**Rust**, Dr. and Prof. of *Vienna*, Treatise on Diseases of the Joints . 183
**Salamanca**, Dr. of *Cadiz*, on the Use of the Prussiate of Mercury . 119
**Salva**, Dr. of *Barcelona*, observations on Materia Medica . . 112
**Schabel**, Dr. of *Weissenbourg*, Experiments on the Effects of Hellebore 118
**Speer**, Dr. of *Bath*, Treatise on the Functions of the Stomach . 235
**Stanley**, Mr. of *London*, Treatise on Anatomy . . . 272
**Stevenson**, Mr. of *London*, Treatise on Ophthalmia . . . 511
**Stoker**, Dr. of *Dublin*, observations on Fever . . 49, 503
Apoplexy . . . . . . . . 321
**Tessier**, Dr. of *Paris*, observations on the Period of Utero-Gestation and Incubation . . . . . . . 537
**Tweedie**, Dr. of *Edinburgh*, cases of Dropsy . . . 536
**Wardrop**, Mr. of *London*, observations on Nævi Materni . 16, 485
**Weizmann**, Dr. of *Bucharest*, observations on Venereal Diseases . 64
**White**, Mr. of *Bath*, on the Treatment of Spasmodic Constriction of the Anus . . . . . . . . . 459
**Wilmot**, Mr. of *Dublin*, case of Psoas Abscess . . . 320
**Wistar**, Dr. **Caspar**, of *Philadelphia*, Description of the Sphenoid Bone . . . . . . . . . 8
**Yeats**, Dr. of *London*, remarks on the Opinions of the Ancients respecting Contagion . . . . . . . 485
**Young**, Mr. **Samuel**, of *London*, Treatise on Cancer . . 83
Remarks on Ligature of the Aorta . . . . . 377

---

**Transactions of Medical Societies.**

Of the Medico-Chirurgical Society of London, Vol. IX. Part I. 88, 146, 156
Of the Association of Fellows and Licentiates of the King's and Queen's College of Physicians, Dublin; Vol. II. . 315, 406, 492

